# Teaching of manual cataract extraction in ophthalmic surgical training programmes

**DOI:** 10.1007/s11845-024-03712-7

**Published:** 2024-05-21

**Authors:** Alexandra McCreery, Amy O’Regan, Noel Horgan

**Affiliations:** 1 St Vincent’s University Hospital, Dublin, Ireland; 2 The Royal Victoria Eye and Ear Hospital, Dublin, Ireland

**Keywords:** Cataract surgery, Ophthalmology, Wet laboratory

## Abstract

**Background:**

Manual cataract extraction techniques such as extracapsular cataract extraction (ECCE) or manual small incision cataract (MSICS) surgery have been replaced by phacoemulsification cataract surgery. Surgical training opportunities for manual techniques of cataract extraction are limited in modern surgical training programmes.

**Aims:**

This study evaluated the current trends of ECCE/MSICS training opportunities amongst trainees and trainers in the Irish Ophthalmic Surgical Training Program.

**Methods:**

An electronic survey was distributed to all ophthalmic surgical trainees and consultants in the country. It addressed the experience and exposure to ECCE/MSICS.

**Results:**

Nineteen of 33 (57%) trainees and 29 of 55 (55%) of consultants completed the survey. Twelve of 19 (63%) trainees viewed an ECCE procedure performed live. Twenty-seven of 29 (93%) consultants performed an ECCE procedure during their surgical career; 8 of 27 (30%) performed an ECCE within the last 1–3 years. Fourteen of 19 (74%) trainees stated they do not feel confident converting from phacoemulsification to ECCE independently. Sixteen of 19 (89%) trainees believe manual cataract extraction training should be included in their surgical training. Nineteen of 29 (65%) consultants believe training in manual cataract extraction should be part of the surgical training programme.

**Conclusions:**

There is a paucity of manual cataract extraction being performed in Ireland, limiting live surgical training in this technique. This survey highlights the limited experience of trainees in this valuable skill that is occasionally required for a successful surgical outcome. The authors conclude that wet laboratory simulated training of manual cataract extraction will bridge this gap.

## Background

Cataract, which is opacification of the crystalline lens in the eye, is the most common cause of visual impairment globally [1]. It may eventually lead to blindness if not treated and carries a significant visual impairment burden for patients. Treatment options are exclusively surgical, involving extraction and replacement of the native lens with an intraocular lens implant. This is a permanent solution and offers restoration of vision. Cataract surgery is the most commonly performed procedure in ophthalmology and is one of the most cost-effective surgical interventions in terms of quality-of-life improvement [2]. Extraction of a cataract can be manual, which involves removal of the cataract material without emulsification, or it may be removed by emulsification of the lens material (phacoemulsification).

Over time, methods of cataract extraction have evolved in synchrony with developing technology. Surgical training in cataract surgery techniques has changed to mirror this shift. Therefore, newer trainees tend to know newer techniques exclusively. For several decades, phacoemulsification has evolved as the predominant method for cataract extraction worldwide, particularly in more economically developed countries. Phacoemulsification involves the creation of a self-sealing wound and use of an ultrasonic probe to divide, emulsify and aspirate the cataractous lens. The advantages of this method are numerous and inarguable. They include safer and faster cataract extraction, reduced risk of post-operative astigmatism [3], lower rates of infection and wound dehiscence [4] and speedier visual recovery for patients. Phacoemulsification is considered the safest and preferred method of cataract surgery in the developed world.

Prior to phacoemulsification, a method of manual cataract extraction called extracapsular cataract extraction (ECCE) predominated and was therefore the method taught to surgical trainees in the past. ECCE typically involves a 12-mm incision with expression of the cataractous lens, leaving an intact posterior capsule and placement of an intraocular lens. It necessitates the use of sutures to close the wound and requires more operative time when compared to phacoemulsification, and post-operative visual recovery is longer. It does not rely on ultrasonic instrumentation and expensive technology unlike phacoemulsification surgery.

Another technique for manual cataract extraction is MSICS (manual small incision cataract surgery) which is a modification of ECCE. The incision is smaller, self-sealing and does not require sutures. Extraction is manual with expression of the lens through the wound, similar to ECCE. MSICS is a technically more challenging procedure and is widely used in developing countries where the removal of sutures proves challenging as patients are often remote from ophthalmic centres [5, 6]. Both phacoemulsification and manual cataract extraction have excellent visual outcomes [7].

In cases of patients with very dense nuclear cataracts, corneal opacification, significant zonular loss or dialysis, or lens subluxation, a planned manual cataract extraction method may be indicated. In cases of intraoperative capsulorhexis complications, ruptured posterior capsules, and lens dislocation into the vitreous, a conversion from phacoemulsification to manual cataract extraction may be necessary. Successful conversion at the time of the primary surgery will avoid the necessity for a second operative procedure.

With the advent of phacoemulsification, surgical training programmes largely exclude manual cataract extraction from the formal curriculum.

## Aims

We aimed to evaluate the current exposure, experience and opinions of trainees and trainers in Ireland regarding manual cataract extraction and its place in formal surgical training programmes. We developed a survey to assess the status of manual cataract extraction including ECCE and MSICS exposure amongst Irish surgical trainees. We wished to assess the extent to which ophthalmologists consider ECCE/MSICS an important skill for trainees to acquire.

## Methods

A survey was designed and tailored for consultants and trainees. After development, its content was approved by three consultant ophthalmologists. An electronic version was distributed to Irish ophthalmologists via the Irish College of Ophthalmologists mailing list which included approximately 340 members of the college. Our survey primarily focused on the manual cataract extraction technique of ECCE with a single question in either survey directed at MSICS specifically.

## Results

Nineteen of 33 (57%) ophthalmic surgical trainees on programme and 29 of 55 (55%) consultant ophthalmic surgeons completed the survey. Of the trainees surveyed, 12 of 19 (63%) had seen an ECCE procedure performed, 5 of 12 (42%) trainees assisted and 7 of 12 (58%) trainees were the primary surgeon. Out of the trainees who witnessed an ECCE procedure, 9 of 12 (75%) were planned, and 3 of 12 (25%) were not. Five of 19 (26%) trainees had exposure to ECCE procedures in wet lab scenarios. Fourteen of 19 (74%) stated they would not feel confident converting from phacoemulsification to ECCE independently if required. Sixteen of 19 (89%) of trainees believe that ECCE procedure training should be included in their formal surgical training. MSICS exposure occurred in 3 of 19 (15%) trainees, with 17 of 19 (89%) trainees stating they believe it should be included in formal training.

Of the consultants surveyed, 15 of 29 (52%) have over 15-year experience as a consultant. Twenty-one of 29 (72%) had performed an ECCE procedure as the primary surgeon, and as a consultant, 6 of 29 (20%) had performed an ECCE as a trainee. In total, 2 of 29 (7%) consultants had not performed an ECCE procedure at any stage in their career as the primary surgeon. Of the 27 consultants remaining who have performed ECCE previously, 9 of 27 (32%) have performed ECCE over 10 years ago (32%). Eight of 27 (30%) consultants have performed an ECCE within 1 to 3 years. The remaining 10 of 27 (37%) consultants performed the surgery with 3 to 10 years.

Twenty-one of 29 (72%) stated they would feel confident converting to ECCE if required, and 27 of 29 (93%) stated the operating theatres they worked in would be equipped to convert from phacoemulsification to ECCE. Nine of 29 (31%) consultants have supervised a trainee in performing an ECCE or MSICS procedure. Six of 29 (21%) consultants have previously performed an MSICS procedure. Nineteen of 29 (65%) consultants believe some form of formal training in ECCE should be part of the surgical training programme.

## Conclusions

Our survey highlights the mirrored shift in training from manual cataract extraction training with the evolution of phacoemulsification dominating cataract surgery. While phacoemulsification has undoubtedly been a great advance in modern cataract surgery, it is important to recognize the continued relevance of manual cataract extraction techniques such as ECCE and MSICS particularly in certain surgical situations. Our results mimic training programme trends in the United States. In a similar survey of trainees, Henderson et al. showed that 91% of their responders believed there are instances where ECCE may be the preferred procedure and reported a 40% decrease in ECCEs being performed by residents from 2005 to 2010. The US residents in this study performed approximately three to four ECCE procedures throughout their training in 2010 [8]. A more recent report of ophthalmic training programmes in the US showed that in 92% of programmes, a wet laboratory component was part of the surgical curriculum [9].

Our survey highlights the lack of experience trainees have regarding ECCE or MSICS and reveals a potential gap in ophthalmic surgical training opportunities. Embracing a comprehensive approach to cataract surgery training should allow for tailored interventions and ensure that end-of-training graduates possess the skillset to deal with less common intraoperative scenarios at the time of cataract surgery. In practice, planned formal teaching for conversion to ECCE is impractical as the need is unpredictable and relatively rare. Therefore, there may be a potential value in wet lab simulated training for trainees.

As with all surveys, this study may not accurately reflect the practice of all ophthalmologists in Ireland or all programme trainers. However, it does reflect the majority opinion that some experience of manual cataract extraction techniques retains importance in ophthalmic surgical training.
